# Clinical epidemiology and outcomes of emergency department-acute kidney injury: A systematic review

**DOI:** 10.1016/j.heliyon.2024.e30580

**Published:** 2024-05-04

**Authors:** Tsz Yan Cheung, Kelvin Lam, Siu Chung Leung, Timothy H. Rainer

**Affiliations:** a Li Ka Shing Faculty of Medicine, The University of Hong Kong, Hong Kong; b Department of Emergency Medicine, Li Ka Shing Faculty of Medicine, The University of Hong Kong, Hong Kong

**Keywords:** Acute kidney injury, Detection, Diagnosis, Emergency department, Epidemiology, Outcome, Systematic review

## Abstract

**Background:**

Over half of all community-acquired acute kidney injury (CA-AKI) initially presented to emergency department (ED), but emergency department acute kidney injury (ED-AKI) is poorly characterised, poorly understood with no systematic review, often under-recognized and under-managed.

**Objective:**

To review the incidence, risk factors, and outcomes of ED-AKI, and risk factors of post-ED-AKI mortality globally.

**Methods:**

We included published prospective or retrospective observational studies, controlled trials, and systematic reviews reporting AKI in adult ED attendees within 24 h of ED admission. Iatrogenic causes of AKI from medical interventions were excluded. We used PubMed to identify articles from 1996 to August 14, 2021, and adopted the National Heart, Lung, and Blood Institute (NHLBI) Quality Assessment Tool for Observational Cohort and Cross-Sectional Studies to assess risk of bias. We used a Forest plot to present pooled ED-AKI incidence rates and I^2^ statistics. Other parameters were summarized narratively.

**Results:**

Using 24 h from ED admission as the definition for ED-AKI we identified six articles from 2005 to 2018 in high-income settings and one article with a 48-h timeframe. The pooled incidence of ED-AKI was 20 per 1000 adult ED attendances. Risk factors for ED-AKI included increasing age, nursing home residence, previous hospital admission within 30 days, discharge diagnosis of diabetes, obstructive uropathy, sepsis, gastrointestinal medical conditions, high serum creatinine, bilirubin, C-reactive protein, white blood cell, alanine aminotransferase, low serum sodium or albumin on admission, poor premorbid renal function, antibiotic use, active malignancy, lung disease, hyperlipidaemia, and infection. Crude, all-cause 24-h mortality rate was 4.56 % and the one-year mortality rate was 35.04 %. Increasing age and comorbidities including cardiovascular disease and malignancy were associated with higher mortality rates.

**Conclusion:**

The review reveals a paucity of relevant literature which calls for further research, increased vigilance, red flag identification, and standardized management protocols for ED-AKI.

## 1 Introduction

### 1.1 Background

Acute kidney injury (AKI) has a worldwide pooled incidence of 216 per 1000 adults patients and a mortality rate of 23.9 % in adults [1]. AKI is associated with multiple long-term sequelae including chronic kidney disease (CKD) and reliance on renal replacement therapy (RRT) [2]. In 2012 AKI posed a significant financial burden with additional cost of US$1795 per admission in the United States whilst from 2010 to 2011 there was an estimated total inpatient expenditure of £1.02 billion in England annually [3,4].

AKI refers to sudden loss of kidney function, characterised by raised serum creatinine and reduced urine output within 7 days [5,6]. Emergency department (ED) AKI (ED-AKI) may be viewed as a sub-set of community acquired-AKI (CA-AKI) or as a subset of hospital AKI, as the ED really sits at the interface of both. ED-AKI is not a period when AKI will develop from non-AKI; rather it is a period in which AKI is diagnosed, and during which it needs appropriate management. The reason why we focus on this phase and context of ED care is because the United Kingdom (UK) ‘National Confidential Enquiry into Patient Outcome and Death’ (NCEPOD) describe this period as neglected, badly managed, poorly documented and with significant preventable mortality and morbidity. Notably, ED-AKI contributes to a significant proportion of CA-AKI with over half of the patients with e-alerts for CA-AKI first presented to ED [16].

However, ED-AKI has no formally agreed definition. In this review, we propose to define ED-AKI as AKI diagnosis within 24 h from ED registration. There is no fixed duration of patients' stay in the ED, which varies across the world from 4 h to several days. We have set an arbitrary figure of 24 h as a pragmatic and functional compromise for the review. Additionally, blood results including follow up tests would normally be completed within this period. A shorter timeframe would be unfeasible for analysis due to the turnover time for blood tests. Moreover, many studies examining AKI in acute hospital settings report the incidence within 24 h of ED attendance although the definition of AKI is also variable. As this definition is not established, and there is no formal agreement on what time limit indicates ‘acute’ or ‘emergency’ we have undertaken a sensitivity analysis also including publications with 48 h reporting.

ED-AKI is poorly characterized, poorly understood, often under-recognized and under-managed. The UK NCEPOD study found that AKI recognition, management, and documentation were suboptimal among AKI patients that died in hospital [7]. This may be partly due to insufficient research and understanding on ED-AKI given that a substantial proportion of AKI cases in the hospital is considered as ED-AKI. Few studies separately analyzed ED-AKI from other CA-AKI, and there has been no systematic review on ED-AKI. Therefore, we performed a review on the epidemiology and outcomes of ED-AKI. This will clarify the global burden ED-AKI's, identify risk factors to prompt ED-AKI suspicion and encourage the emergency medicine community to treat this syndrome seriously and actively. We raise issues of AKI definition to encourage debate, clarity and uniformity of reporting. The review may help develop strategies to prevent complications and reduce mortality in susceptible groups.

### 1.2 Objectives

Our main objectives were firstly, to describe the incidence and demographics of ED-AKI; secondly, to identify the common risk factors; thirdly, to describe post-ED-AKI outcomes; and finally, to describe risk factors for ED-AKI mortality. Important outcomes include rate of hospital admission, and length of stay in hospital; Intensive Care Unit (ICU) admission and length of stay; in-hospital and intensive care unit (ICU) 30-day, 1-year and 3-year mortality; RRT, and progression to CKD.

## 2 Methods

This systematic review is reported in accordance with the Preferred Reporting Items for Systematic reviews and Meta-Analyses (PRISMA) 2020 statement [8]. The study protocol has been published in the International Prospective Register of Systematic Reviews (PROSPERO, CRD42021281851).

### 2.1 Study eligibility criteria

We included published prospective or retrospective observational studies, controlled trials, and systematic reviews reporting AKI in adult (aged 18 years or above) ED attendees of all presentations. Case reports and studies with only patients under 18 years old, or patients who did not present to the ED, or iatrogenic cause of AKI from the medical interventions (drug, intravenous contrast media) were excluded.

### 2.2 Search strategy

The PubMed database (National Centre for Biotechnology Information, U.S. National Library of Medicine, National Institutes of Health, United States) was used for screening papers from 1996 to the present. The search was run on August 14, 2021. A librarian from The University of Hong Kong advised on the search strategy (Supplementary 1). Only one database was used due to time and resource limitation.

### 2.3 Study selection and data extraction

LK and CTY independently screened titles and abstracts of all articles identified by search. Any discrepancy was discussed and resolved among four independent reviewers (TR, SCL, LK, and CTY). This process was repeated for screening of full text of shortlisted articles. LK and CTY extracted study characteristics including study design, time period, country, sample size, AKI definition, AKI timeframe from ED attendance, determination of baseline creatinine, and data as reported in the objectives. As part of sensitivity analysis, studies reporting on AKI diagnosis within 48 h of ED admission were also included for comparison.

### 2.4 Risk of bias assessment

The National Heart, Lung, and Blood Institute (NHLBI) Quality Assessment Tool for Observational Cohort and Cross-Sectional Studies was used to assess the risk of bias to ascertain internal validity [9,10]. This applies to cohort and cross-sectional studies. Papers were graded as good, fair, or poor. LK and CTY assessed articles independently and four reviewers discussed discrepancies.

### 2.5 Data synthesis and analysis

A Forest plot was created on R with random-effects model to summarize the incidences, 95 % confidence intervals, and heterogeneity by I^2^ statistics [11]. Although we initially intended to conduct a systematic review and meta-analysis, there was insufficient material for such an analysis, so we adopted a narrative review [12].

## 3 Results

**Fig. 1:**
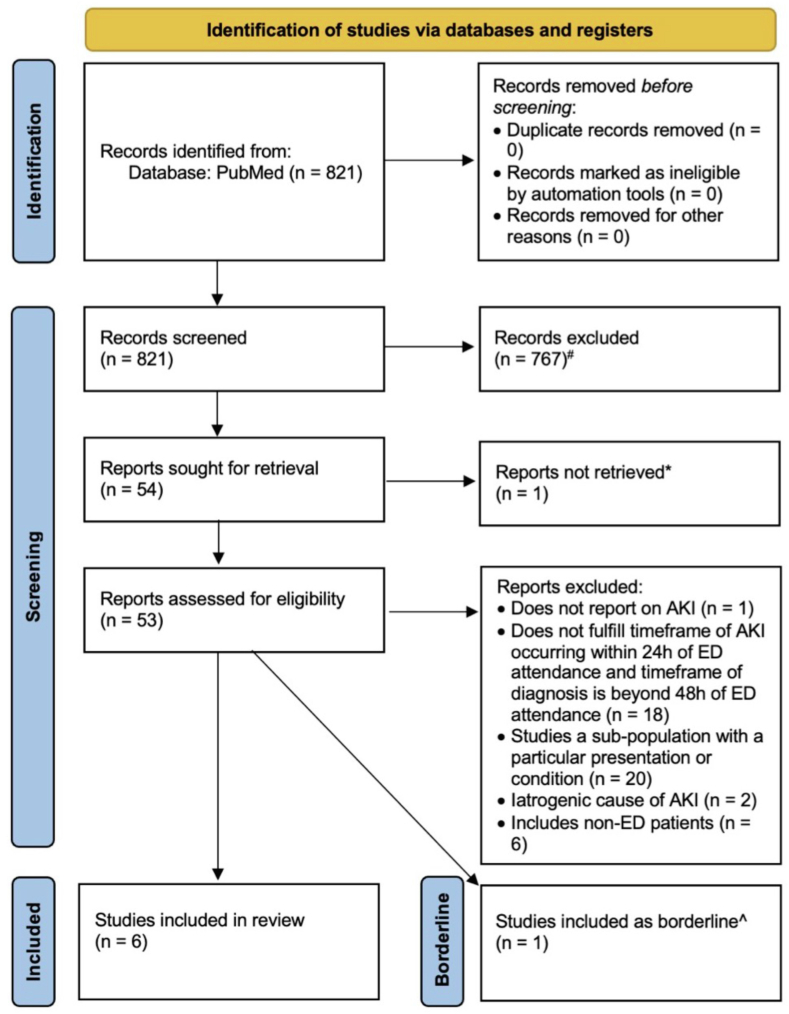
Flow diagram for selection of studies.

The initial search yielded 821 records. Fig. 1 shows the selection process flow including exclusion reasons. After screening of titles and abstracts, 54 articles remained for full-text screening, of which six articles were included [[13], [14], [15], [16], [17], [18]]. One article with AKI diagnosis within 48 h of ED admission was identified [19]. Its results were reviewed and compared with the six papers that met criteria of AKI diagnosis within 24 h.

### 3.1 Descriptive characteristics

Study characteristics are summarized in Table 1. All six studies (4 cohort studies, 1 cross-sectional study and 1 case-crossover study) were conducted in high-income countries from 2005 to 2018 (Table 1) [[13], [14], [15], [16], [17], [18]]. The Kidney Disease Improving Global Outcomes (KDIGO) criteria was most often used for AKI definition [13,14,17,18].

**Table 1:** Characteristics of included studies.

Reference	Study Design/Source of Data	Year	Country	Sample Size	AKI Definition	Setting of AKI Diagnosis	Baseline Creatinine	Exclusion of CKD/Severe AKI/Dialysis Patients
[19]	Cross-sectional study	2017–2018	Switzerland	65489	Acute kidney injury network classification (AKIN 1–3) (urine output criteria not used)	In ED	Pre-admission creatinine or ADQI^#^ back formula for baseline creatinine level calculation	None
[16]	Retrospective cohort + Case-control	2016–2017	UK	20421	KDIGO sCr component	In ED/Acute medical admission ward	Historic sCr or extrapolation from admission and post-discharge sCr	None
[18]	Case-crossover study with retrospective data	2013–2015	Australia	1815^c^	ICD 10 Code in the Emergency Department Information System	In ED	Method for derivation of baseline creatinine is unclear in the article	None
[17]	Prospective observational study	2013	Switzerland	8464	KDIGO sCr component	In ED	Historic sCr or the lowest creatinine value within the next 28 days after AKI	Excludes patients already on chronic dialysis or with 2 admissions during screening period
[20]	Retrospective Cohort	2005–2014	Ireland	87193	KDIGO sCr component	In ED/GP/IP/OP^a^	Median creatinine value within 3 months prior to AKI/median creatinine values recorded within 3 months following AKI/minimum creatinine value within 48 h following AKI	Excludes patients with kidney failure receiving dialysis
[21]	Retrospective study	2010	Iceland	53816	KDIGO sCr component	In ED		None
Borderline Paper^b^								
(22)	Prospective observational	2007	Portugal	616	Risk Injury Failure Loss End-Stage Kidney Disease and Acute Kidney Injury Network criteria: New increase in SCr that did not resolve in 72 h.	In ED/IP	Historic SCr from 1 to 6 months before ED presentation	Excludes non-hospitalized patients, patients with stablished CKD stage 4, severe AKI at admission (increase in SCr >300 % over baseline or >4 mg/dl for >48 h or needing)

a Paper looks at AKI from a broad spectrum of settings (inpatient facility (IP), outpatient facility (OP), general practice (GP)), but only the ED group is included in the review.

b Samples used for AKI diagnosis were taken up to 48 h after ED arrival.

c Number of ED-AKI cases.

### 3.2 Incidence of ED-AKI

**Fig. 2:**
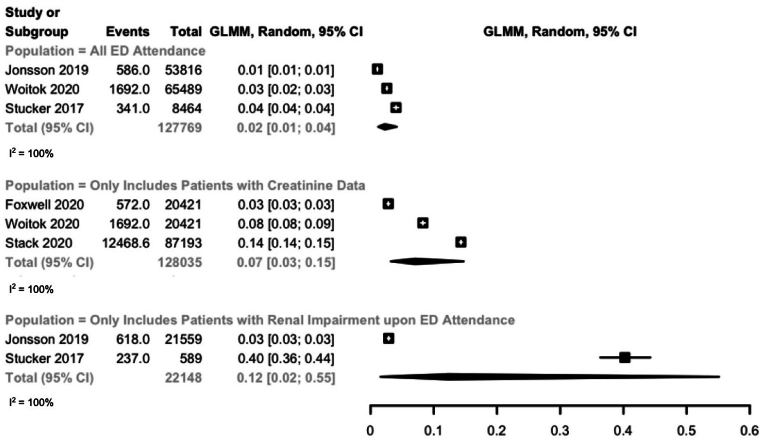
Incidence of emergency department-acute kidney injury by population group.

Five studies reported on the incidence of ED-AKI [13,14,[16], [17], [18]]. They are reported narratively with separate summary statistics for each population group (Fig. 2). Jonsson, Woitok, and Stucker's papers are included in more than one population group since they presented incidence for multiple population groups separately. The Incidence of ED-AKI ranged from 10 to 40 per 1000 adult ED attendance with a combined incidence of 20 per 1000 adult ED attendance (95 % CI 0.0.1, 0.04). Among ED attendees with an available creatinine test, combined incidence was 70 per 1000 adult ED attendance (95 % CI 0.03, 0.15). For ED attendees with renal impairment revealed by elevated serum creatinine levels or reduced estimated glomerular filtration rate (eGFR) on admission, combined incidence was 120 per 1000 adult ED attendance (95 % CI 0.02, 0.55). The incidence of ED-AKI was heterogeneous among all population groups with an I^2^ value of 100 % for all groups.

### 3.3 Risk factors of ED-AKI

Four studies analyzed associations between characteristics at ED admission and ED-AKI (Table 2). For demographics, there was strong evidence that increased age (p < 0.01 for all studies), nursing home residents (RR: 1.64, p < 0.001), patients with previous hospital admission within 30 days (RR: 1.21, p = 0.018) and higher environmental temperature (RR: 1.36) were associated with a higher risk of ED-AKI. Results on sex was inconsistent with two studies showing an increased risk of ED-AKI among males [13,14], and another study showing reduced risk in males [18]. For admission laboratory tests, higher serum creatinine, bilirubin, C-reactive protein, white blood cell, alanine aminotransferase (ALT), and lower serum sodium, and albumin were associated with higher risks of ED-AKI (p < 0.01). Patients discharged with diabetes, urological obstruction, sepsis, and gastrointestinal medical condition were more likely to have ED-AKI, whereas patients with trauma and other general medical/surgical conditions were less likely to have ED-AKI.

**Table 2:** Association between ED-AKI and characteristics on ED admission.

	Reference		Total No. (%)	No. with ED-AKI (%)	No. without ED-AKI (%)	RR (95%CI)	p-value
**Demographics**							
Age (mean ± SD)	[16]	–	–	70.3±18.0	54.7±21.8	–	<0.001
	[17]	–	–	72.8 ± 15.4	79.7±11.4	–	0.001
	[21]	–	–	67.5 (18–98)	69.7 (18–104)	–	0.01
Male	[16]	Male	565 (50.49 %)	301 (53.27 %)	264 (46.73 %)	1.19 (1.07, 1.32)	0.004
		Female	554 (49.51 %)	247 (44.58 %)	307 (55.42 %)		
	[17]	Male	310.39 (53.61 %)	198.53 (63.96 %)	111.86 (36.04 %)	1.21 (1.07, 1.35)	0.02
		Female	268.61 (46.39 %)	142.47 (53.04 %)	126.14 (46.96 %)		
	[21]	Male	1740 (60.46 %)	329 (18.91 %)	1411 (81.09 %)	0.84 (0.69, 0.98)	0.02
		Female	1138 (39.54 %)	257 (22.58 %)	881 (77.42 %)		
Nursing/Residential Home Resident	[16]	Nursing/Residential Home Resident	144 (12.87 %)	107 (74.31 %)	37 (25.69 %)	1.64 (1.52, 1.76)	<0.001
		Non-Nursing/Residential Home Resident	975 (87.13 %)	441 (45.23 %)	534 (54.77 %)		
Previous Hospital Admission within 30 days	[16]	Previous Hospital Admission	178 (15.91 %)	102 (57.30 %)	76 (42.70 %)	1.21 (1.07, 1.35)	0.018
		No Previous Hospital Admission	941 (84.09 %)	446 (47.40 %)	495 (52.60 %)		
Environmental Temperature	[18]	–	–	–	–	1.37 (1.10, 1.71)	–
Laboratory Tests on Admission (mean ± SD)							
Creatinine (μmol/L)	[16]	–	–	190.5±117.6	78.09±27.9		<0.001
Sodium (mmol/L)	[16]	–	–	135.3±6.6	137.6±4.0		<0.001
Albumin (g/L)	[16]	–	–	31.1±6.7	36.1±6.0		<0.001
Bilirubin (μmol/L)	[16]	–	–	22.0±43.5	12.6±19.0		<0.001
C-reactive protein (mg/L)	[16]	–	–	107.3±114.9	29.9±54.9		<0.001
White blood cell (10^9/L)	[16]	–	–	12.8±7.2	9.7±4.6		<0.001
Alanine transaminase (U/L)	[16]	–	–	58.2±161.0	34.9±90.9		0.005
Discharge Diagnosis							
Diabetes	[16]	Diabetes	31 (2.77 %)	30 (96.77 %)	1 (3.23 %)	2.03 (1.94, 2.12)	<0.001
		No Diabetes	1088 (97.23 %)	518 (47.61 %)	570 (52.39 %)		
Urological Obstruction	[16]	Urological Obstruction	64 (5.72 %)	57 (89.06 %)	7 (10.94 %)	1.91 (1.81, 2.02)	<0.001
		No Urological Obstruction	1055 (94.28 %)	491 (46.54 %)	564 (53.46 %)		
Sepsis	[16]	Sepsis	264 (23.59 %)	187 (70.83 %)	77 (29.17 %)	1.68 (1.57, 1.79)	<0.001
		No Sepsis	855 (76.41 %)	361 (42.22 %)	494 (57.78 %)		
Trauma	[16]	Trauma	91 (8.13 %)	26 (28.57 %)	65 (71.43 %)	0.56 (0.23, 0.89)	<0.001
		No Trauma	1028 (91.87 %)	522 (50.78 %)	506 (49.22 %)		
Medical/Surgical General	[16]	Medical/Surgical General	302 (26.99 %)	25 (8.28 %)	277 (91.72 %)	0.13 (−0.25, 0.51)	<0.001
		No Medical/Surgical General	817 (73.01 %)	523 (64.01 %)	294 (35.99 %)		
Gastrointestinal Medical	[16]	Gastrointestinal Medical	108 (9.65 %)	72 (66.67 %)	36 (33.33 %)	1.42 (1.27, 1.56)	0.002
		No Gastrointestinal Medical	1011 (90.35 %)	476 (47.08 %)	535 (52.92 %)		
Surgical Emergency	[16]	Surgical Emergency	59 (5.27 %)	38 (64.41 %)	21 (35.59 %)	1.34 (1.14, 1.54)	0.29
		No Surgical Emergency	1060 (94.73 %)	510 (48.11 %)	550 (51.89 %)		
Cardiovascular	[16]	Cardiovascular	115 (10.28 %)	59 (51.30 %)	56 (48.70 %)	1.05 (0.86, 1.24)	1
		No Cardiovascular	1004 (89.72 %)	489 (48.71 %)	515 (51.29 %)		
Malignancy	[16]	Malignancy	25 (2.23 %)	14 (56.00 %)	11 (44.00 %)	1.15 (0.79, 1.50)	1
		No Malignancy	1094 (97.77 %)	534 (48.81 %)	560 (51.19 %)		

Three studies analyzed associations between medical and drug history with ED-AKI (Table 3). Patients with higher pre-admission mean creatinine, lower baseline estimated glomerular filtration rate (eGFR), and chronic kidney disease were associated with higher risks of ED-AKI (p < 0.001 for all). Those with a prior history of AKI had 1.26 times risk of ED-AKI (p = 0.046). Foxwell found that patients with lung disease had higher risk of ED-AKI (RR: 2.12, 95%CI: 2.04, 2.20, p < 0.001). ED-AKI risk was also increased among patients with hyperlipidemia and infections. Evidence on malignancy, cardiac disease, hypertension, diabetes mellitus, and cirrhosis were inconsistent among the studies. For medications, Stucker found that antibiotics were associated with a higher risk of ED-AKI (RR: 1.31, 95%CI: 1.05, 1.56, p = 0.04).

**Table 3:** Association between emergency department-acute kidney injury and premorbid conditions & drug history.

		Reference		Total No. (%)	No. with ED-AKI (%)	No. without ED-AKI (%)	RR (95%CI)	p-value
Premorbid Renal Condition								
Mean Creatinine (pre-admission) ± SD (μmol/L)		[16]	–	–	94.5±42.7	77.0±26.8	–	<0.001
Baseline eGFR (pre-admission for Foxwell), mean ± SD		[16]	–	–	81.0±31.0	101.7±29.0	–	<0.001
		[21]	–	–	69.76	48.03	–	<0.0001
Chronic Kidney Disease	Pre-admission CKD 3a-5	[16]	Pre-admission CKD 3a-5	196 (17.52 %)	151 (77.04 %)	45 (22.96 %)	1.79 (1.67, 1.90)	<0.001
			No Pre-admission CKD 3a-5	923 (82.48 %)	397 (43.01 %)	526 (56.99 %)		
Prior History of AKI		[21]	Prior AKI	292 (10.15 %)	73 (25.00 %)	219 (75.00 %)	1.26 (1.05, 1.47)	0.046
			No Prior AKI	2586 (89.85 %)	513 (19.84 %)	2073 (80.16 %)		
Comorbid Conditions								
Malignancy	Active Malignancy	[16]	Active Malignancy	93 (8.31 %)	85 (91.40 %)	8 (8.60 %)	2.03 (1.93, 2.12)	<0.001
			No Active Malignancy	1026 (91.69 %)	463 (45.13 %)	563 (54.87 %)		
	Malignancy/Cancer	[17]	Malignancy/Cancer	61.58 (10.64 %)	42.54 (69.08 %)	19.04 (30.92 %)	1.20 (1.01, 1.38)	0.09
			No Malignancy/Cancer	517.42 (89.36 %)	298.46 (57.68 %)	218.96 (42.32 %)		
		[21]	Malignancy/Cancer	531 (18.45 %)	120 (22.60 %)	411 (77.40 %)	1.14 (0.96, 1.32)	0.18
			No Malignancy/Cancer	2347 (81.55 %)	466 (19.86 %)	1881 (80.14 %)		
Peripheral Vascular Disease	Peripheral Vascular Disease	[16]	Peripheral Vascular Disease	159 (14.21 %)	29 (18.24 %)	130 (81.76 %)	0.34 (0.00, 0.67)	0.003
			No Peripheral Vascular Disease	960 (85.79 %)	519 (54.06 %)	441 (45.94 %)		
Ischaemic Heart Failure	Cardiovascular Disease	[16]	Cardiovascular Disease	368 (32.89 %)	196 (53.26 %)	172 (46.74 %)	1.14 (1.01, 1.26)	<0.001
			No Cardiovascular Disease	751 (67.11 %)	352 (46.87 %)	399 (53.13 %)		
	Coronaropathy	[17]	Coronaropathy	147.61 (25.49 %)	80.97 (54.85 %)	66.64 (45.15 %)	0.91 (0.74, 1.08)	0.12
			No Coronaropathy	431.39 (74.51 %)	260.03 (60.28 %)	171.36 (39.72 %)		
Heart Failure	Cardiac Insufficiency	[17]	Cardiac Insufficiency	98.18 (16.96 %)	48.2 (49.09 %)	49.98 (50.91 %)	0.81 (0.59, 1.02)	0.03
			No Cardiac Insufficiency	480.82 (83.04 %)	292.8 (60.90 %)	188.02 (39.10 %)		
	Ischemic Heart Disease	[21]	Ischemic Heart Disease	1062 (36.90 %)	241 (22.69 %)	821 (77.31 %)	1.19 (1.05, 1.34)	0.02
			No Ischemic Heart Disease	1816 (63.10 %)	345 (19.00 %)	1471 (81.00 %)		
Cerebrovascular Disease	Cerebrovascular Disease	[21]	Cerebrovascular Disease	401 (13.93 %)	89 (22.19 %)	312 (77.81 %)	1.11 (0.91, 1.31(	0.36
			No Cerebrovascular Disease	2477 (86.07 %)	497 (20.06 %)	1980 (79.94 %)		
Hypertension	Hypertension	[16]	Hypertension	346 (30.92 %)	288 (83.24 %)	58 (16.76 %)	2.47 (2.36, 2.58)	<0.001
			No Hypertension	773 (69.08 %)	260 (33.64 %)	513 (66.36 %)		
		[17]	Hypertension	323.38 (55.85 %)	187.72 (58.05 %)	135.66 (41.95 %)	0.97 (0.83, 1.10)	0.23
			No Hypertension	255.62 (44.15 %)	153.28 (59.96 %)	102.34 (40.04 %)		
		[21]	Hypertension	1655 (57.51 %)	372 (22.48 %)	1283 (777.52 %)	1.28 (1.13, 1.44)	0.02
			No Hypertension	1223 (42.49 %)	214 (17.50 %)	1009 (82.50 %)		
DM	Diabetes	[16]	Diabetes	255 (22.79 %)	154 (60.39 %)	101 (39.61 %)	1.32 (1.20, 1.45)	<0.001
			No Diabetes	864 (77.21 %)	394 (45.60 %)	470 (54.40 %)		
		[17]	Diabetes	248.51 (42.92 %)	198.53 (79.89 %)	49.98 (20.11 %)	1.85 (1.71, 1.99)	0.08
			No Diabetes	330.49 (57.08 %)	142.47 (43.11 %)	188.02 (56.89 %)		
		[21]	Diabetes	487 (16.92 %)	107 (21.97 %)	380 (78.03 %)	1.10 (0.91, 1.28)	0.37
			No Diabetes	2391 (83.08 %)	479 (20.03 %)	1912 (79.97^)		
Pulmonary Disease	Lung Disease	[16]	Lung Disease	96 (8.58 %)	91 (94.79 %)	5 (5.21 %)	2.12 (2.04, 2.20)	<0.001
			No Lung Disease	1023 (91.42 %)	457 (44.67 %)	566 (55.33 %)		
Liver Disease	Liver Disease	[16]	Liver Disease	52 (4.65 %)	29 (55.77 %)	23 (44.23 %)	1.15 (0.90, 1.40)	<0.001
			No Liver Disease	1067 (95.35 %)	519 (48.64 %)	548 (51.36 %)		
	Cirrhosis	[17]	Cirrhosis	10.24 (1.77 %)	7.86 (76.76 %)	2.38 (23.24 %)	1.31 (0.97, 1.65)	0.54
			No Cirrhosis	568.76 (98.23 %)	333.14 (58.57 %)	235.62 (41.43 %)		
Others	Hyperlipidaemia	[16]	Hyperlipidaemia	194 (17.34 %)	179 (92.27 %)	15 (7.73 %)	2.31 (2.22, 2.40)	<0.001
			No Hyperlipidaemia	925 (82.66 %)	369 (39.89 %)	556 (60.11 %)		
	Connective Tissue Disease/Vasculitis	[16]	Connective Tissue Disease/Vasculitis	67 (5.99 %)	28 (41.795)	39 (58.21 %)	0.85 (0.56, 1.13)	0.42
			No Connective Tissue Disease/Vasculitis	1052 (94.01 %)	520 (49.43 %)	532 (50.57 %)		
	Dementia	[16]	Dementia	98 (8.76 %)	59 (60.20 %)	39 (39.80 %)	1.26 (1.08, 1.43)	<0.001
			No Dementia	1021 (91.24 %)	489 (47.89 %)	532 (52.11 %)		
	Infection	[17]	Infection	42.87 (7.40 %)	33.35 (77.79 %)	9.52 (22.21 %)	1.36 (1.18, 1.53)	0.01
			No Infection	536.13 (92.60 %)	307.65 (57.38 %)	228.48 (42.62 %)		
Medications								
RAA Blockers		[17]	RAA Blockers	266.06 (45.95 %)	154.2 (57.96 %)	111.86 (42.04 %)	0.97 (0.83, 1.11)	–
			No RAA Blockers	312.94 (54.05 %)	186.8 (59.69 %)	126.14 (40.31 %)		
ARBs		[17]	ARBs	156.51 (27.03 %)	89.87 (57.42 %)	66.64 (42.58 %)	0.97 (0.81, 1.12)	–
			No ARBs	422.49 (72.97 %)	251.13 (59.44 %)	171.36 (40.56 %)		
ACEIs		[17]	ACEIs	109.31 (18.88 %)	52.19 (47.74 %)	57.12 (52.26 %)	0.78 (0.57, 0.99)	–
			No ACEIs	469.69 (81.12 %)	288.81 (61.49 %)	180.88 (38.51 %)		
Diuretics		[17]	Diuretics	233.27 (40.29 %)	138.07 (59.19 %)	95.2 (40.81 %)	1.01 (0.87, 1.15)	–
			No Diuretics	345.73 (59.71 %)	202.93 (58.70 %)	142.8 (41.30 %)		
RAAB + Diuretics		[17]	RAAB + Diuretics	146.08 (25.23 %)	86.58 (59.27 %)	59.5 (40.73 %)	1.01 (0.85, 1.16)	0.99
			No RAAB + Diuretics	432.92 (74.77 %)	254.42 (58.77 %)	178.5 (41.23 %)		
NSAID		[17]	NSAID	26.56 (4.59 %)	19.42 (73.12 %)	7.14 (26.88 %)	1.26 (1.01, 1.50)	0.37
			No NSAID	552.44 (95.41 %)	321.58 (58.21 %)	230.86 (41.79 %)		
Antibiotics		[17]	Antibiotics	20.02 (3.46 %)	15.26 (76.22 %)	4.76 (23.78 %)	1.31 (1.05, 1.56)	0.04
			No Antibiotics	558.98 (96.54 %)	325.74 (58.27 %)	233.24 (41.73 %)		

### 3.4 Outcomes of ED-AKI

The 24-h mortality was 4.56 % (95%CI 2.81 %, 6.31 %), in-hospital mortality was 25.00 % (95%CI 21.37 %, 28.63 %), and 1-year mortality up to 35.04 % (31.04 %, 39.03 %) (Table 4). Only 65.18 % of ED-AKI patients (95 % CI 49.90 %, 70.45 %) had recovery of renal function following ED-AKI. 83.76 % ED-AKI patients were hospitalized, and mean length of stay was 10 days.

**Table 4:** Outcomes of emergency department-acute kidney injury.

	Reference	Sample Size	No. with Outcome	Proportion (95 % CI)
Mortality				
24h Mortality	[16]	548	25	4.56 % (2.81 %, 6.31 %)
72h Mortality	[16]	548	68	12.41 % (9.65 %, 15.17 %)
7-day Mortality	[16]	548	133	24.27 % (20.68 %, 27.86 %)
In-hospital Mortality	[16]	548	137	25.00 % (21.37 %, 28.63 %)
90-day Post-hospital Discharge Mortality	[16]	548	155	28.28 % (24.51 %, 32.06 %)
1-year Mortality	[16]	548	192	35.04 % (31.04 %, 39.03 %)
Post-discharge 1-year Mortality if survive to discharge	[16]	548	167	30.47 % (26.62 %, 34.33 %)
Renal Outcome				
AKI Progression	[16]	548	80	14.60 % (11.64 %, 17.55 %)
Recovery of Renal Function	[17]	313	204	65.18 % (59.90 %, 70.45 %)
Hospital Admission-related Outcomes				
Hospital Admission	[16]	548	459	83.76 % (80.67 %, 86.85 %)
ICU Admission	[16]	548	45	8.21 % (5.91 %, 10.51 %)
	[17]	341	49	14.37 % (10.65 %, 18.09 %)
30d Hospital readmission	[16]	–	–	20.40 %
Length of Hospital Stay (mean, median & IQR)	[17]	–	–	10 (4–27)
1-year Hospital Days (mean, median & IQR)	[17]	–	–	23 (7–56)

### 3.5 Risk factors for ED-AKI mortality

Increasing age was as a risk factor for mortality in both studies and the odds ratio was 1.029 (95 % CI 1.012, 1.047, p = 0.001) and 1.04 (95%CI 1.01, 1.06, p = 0.001) for in-hospital mortality and 3-year mortality respectively (Table 5). Higher serum albumin and longer length of hospital stay were associated with lower in-hospital mortality. Ischaemic heart disease and hypertension were associated with higher risk for 3-year mortality but not in-hospital mortality. Longer length of hospital stay was associated with reduced in-hospital mortality.

**Table 5:** Risk factors for mortality in patients with emergency department-acute kidney injury.

	Reference	HR/OR (95%CI)	p-value
Hospital Survival			
Demographics			
Age	[16]	1.029 (1.012, 1.047)^a^	0.001
Nursing Home/Residential Home Resident	[16]	1.182 (0.717, 1.946)^a^	0.512
Male vs Female	[16]	0.967 (0.674, 1.388)	0.857
Comorbidities			
Hyperlipidaemia	[16]	0.660 (0.436, 1.001)	0.051
Malignancy	[16]	1.530 (0.970, 2.414)	0.067
Liver Disease	[16]	1.457 (0.779, 2.726)	0.239
Cardiovascular Disease	[16]	0.856 (0.589, 1.243)	0.413
Diabetes	[16]	0.834 (0.539, 1.290)	0.415
Hypertension	[16]	0.889 (0.618, 1.277)	0.524
Lung Disease	[16]	0.684 (0.571, 1.445)	0.684
CKD Evidence	[16]	1.089 (0.668, 1.776)^a^	0.733
Connective Tissue Disease/Vasculitis	[16]	1.120 (0.514, 2.441)^a^	0.776
Dementia	[16]	1.101 (0.692, 1.752)	0.684
Peripheral Vascular Disease	[16]	1.021 (0.497, 2.100)	0.954
Lab Test			
Albumin	[16]	0.946 (0.907, 0.986)^a^	0.008
Sodium	[16]	1.026 (0.999, 1.054)	0.061
ALT	[16]	1.001 (1.000, 1.001)^a^	0.218
White Blood Cell Count	[16]	1.016 (0.990, 1.043)^a^	0.223
Bilirubin	[16]	1.002 (0.999, 1.005)	0.252
C-reactive Protein	[16]	1.000 (0.998, 1.002)^a^	0.938
Parameters related to Hospital Stay			
Length of Stay	(16)	0.004 (0.000, 0.083)^a^	<0.001
ICU Admission	(16)	1.257 (0.717, 2.204)	0.452
3-year Mortality			
Age	[17]	1.04 (1.01, 1.06)	0.001
Heart Disease	[17]	2.70 (1.57, 4.64)	0.001
Malignancy	[17]	2.41 (1.07, 5.45)	0.034
Hypertension	[17]	1.80 (1.01, 3.20)	0.045

a Adjusted Odds Ratio/Hazards Ratio.

### 3.6 Sensitivity analysis

In the study reporting on AKI diagnosis within 48 h of ED admission, the incidence of ED-AKI was 211.0 per 1000 adult ED admissions (95%CI: 17.88 %, 24.33 %) [19]. The risk of ED-AKI increased with age (p < 0.0001), plasma neutrophil gelatinase-associated lipocalin (pNGAL) at 12 h following index admission (p < 0.001), higher baseline serum creatinine, lower baseline eGFR, and chronic kidney disease (p < 0.001), cardiovascular disease, heart failure, hypertension, diabetes, and higher Charlson Comorbidity Index Score (p < 0.001). There was no gender difference.

Outcomes were reported with a median follow-up of 62 months. The overall mortality was 44.62 % (95%CI: 36.07 %, 53.16 %). Out of the 73.85 % who progressed to CKD (95%CI: 66.29 %, 81.40 %), 10.77 % (95%CI: 5.44 %, 16.10 %) required RRT. 13.85 % required ICU admission (95%CI: 7.91 %, 19.78 %) which was comparable to the included studies.

### 3.7 Risk of biased results/methodological quality

Two papers were rated as good quality, and the remaining four papers were fair. The main reasons for fair quality were lack of adjustment for confounders, high loss to follow-up, and lack of reporting on variance or effect estimates (supplementary table 1).

## 4 Discussion

### 4.1 Principal findings

In this systematic review including six studies across five countries, we summarize the incidence, risk factors and outcomes of ED-AKI, as well as risk factors of mortality. To our knowledge, this is the first study to review the literature on the epidemiology and outcomes of ED-AKI, as defined above.

The combined incidence of ED-AKI was 20 per 1000 adult ED admissions, and results were highly heterogeneous with an I^2^ value of 100 %. Possible explanations include the variable AKI definitions adopted, e.g. KDIGO AKI definition and ICD codes, variable patient demographics, and different inclusion and exclusion criteria [20]. Additionally, when the timeframe for ED-AKI diagnosis was increased to 48 h, the incidence increased 10-fold to 211.0 per 1000 adult ED attendances.

Multiple important risk factors for ED-AKI include increased age, nursing home residency, previous hospital admission within 30 days, higher environmental temperature, serum creatinine, bilirubin, C-reactive protein, white blood cell, alanine aminotransferase (ALT), and lower serum sodium and albumin; diabetes, urological obstruction, sepsis, and gastrointestinal medical conditions, higher pre-admission mean creatinine, lower baseline eGFR, CKD, prior history of AKI, lung disease, hyperlipidemia, infections and antibiotics use. These risk factors for ED-AKI include indicators of poorer renal reserve which itself may reduce the threshold for AKI. The elevated white blood cell count, and C-reactive protein may reflect infection and inflammation. For antibiotics, the reviewed study did not differentiate the different classes which may have different nephrotoxicity, and the association may again be confounded by infection. Also, patients taking antibiotics tend to have an infection which is in itself a risk factor for AKI and a potential confounder [5]. The findings on coronary artery disease, congestive heart failure, and diabetes mellitus were variable. This may be due to a lack of subgroup analysis, e.g. differentiation of single and triple vessel disease, different severity of congestive heart failure as reflected by the New York Heart Association classification, insulin dependence and complications in diabetes mellitus. These risk factors often apply to non-ED-AKI as well.

ED-AKI had poor outcomes with a high mortality of 4.56 % in 24-h and 35.04 % in 1 year. ED-AKI also has a high risk of complications including progression to CKD, requirement of RRT, ICU admission, and prolonged hospital stay. These would negatively impact patients’ quality of life and pose a high economic burden on the healthcare system.

While advanced age was a risk factor for ED-AKI in-hospital and long-term mortality, comorbidities including cardiovascular disease and malignancy were only associated with 3-year mortality. Foxwell found that longer length of hospital stay is associated with lower in-hospital mortality, thus suggesting that patients who died from ED-AKI tended to die early on [21].

### 4.2 Comparison with previous studies

To our knowledge, this is the first review on epidemiology ED-AKI. Thus, the findings from this review are compared with previous studies on AKI and CA-AKI. Notably, the number of studies included in this review is much fewer compared to reviews on AKI and CA-AKI since there is limited literature on ED-AKI [1,22,23].

ED-AKI's combined incidence was 20 per 1000 adult ED admissions, compared to AKI's pooled incidence of 216 per 1000 adult patients reported in another systematic review [1]. Most studies included in that systematic review on AKI were conducted in hospitalized or critically ill patients, so the incidence may be overestimated, and CA-AKI may be under-represented. For CA-AKI, the existing literature on the incidence is limited. In the first formal analysis on CA-AKI performed by Kaufman et al., the prevalence of CA-AKI was 1 in 100 hospital admissions [22,24]. Comparing incidences of ED-AKI with overall AKI, a considerable proportion of AKI first presented to the ED. The incidence of ED-AKI using an expanded definition of 48 h after ED presentation was 221 per 1000 adult ED admissions, which approached the pooled incidence rate of AKI in hospitalized patients. This shows that most AKI patients developed AKI within the first 48 h of admission. Heterogeneity of incidence was also seen in the systematic review on AKI.

Similar to previous studies on CA-AKI, higher age and poorer premorbid renal function were found to be risk factors for CA-AKI [22]. Unlike prior studies which reported an increased risk of CA-AKI among patients with coronary artery disease, congestive heart failure, and diabetes mellitus, the current review found conflicting results regarding their association with ED-AKI [22]. For medications, previous research showed medications that negatively affect kidney hemodynamics would increase CA-AKI risk, e.g. non-steroidal anti-inflammatory drugs, angiotensin-converting-enzyme inhibitors, angiotensin II receptor blockers [22]. However, our review showed that only antibiotic use was associated with ED-AKI. It should be noted that there was only one study examining the association between medications and ED-AKI, so more research is needed to support this finding. Additionally, the current review found risk factors that have not been reported in other reviews: nursing home residence, previous hospital admission, active malignancy, lung disease, hyperlipidemia, infection, and discharge diagnosis of diabetes, obstructive uropathy, sepsis, and gastrointestinal medical conditions. For lung disease, Mesropian's review reported an association between community-acquired pneumonia and CA-AKI [22]. It remains unclear whether the differences in risk factors from CA-AKI are due to a genuine difference or the limited sample size for research on ED-AKI.

Regarding ED-AKI outcomes, the 24-h mortality of 4.56 % with one-year mortality up to 35.04 % was much higher than AKI's pooled mortality rate of 27.7 % when patients were followed-up for more than 6 months [1]. It may be due to a lack of diagnostic vigilance and timely intervention at the ED when compared to in-hospital care, different underlying disease mechanisms, e.g. more trauma-related causes at the ED, and premorbid conditions. Other outcomes of ED-AKI were similar [1].

The risk factors for ED-AKI mortality were advanced age, and comorbidities (cardiovascular disease and malignancy). These were also found to be risk factors of mortality in a previous review on AKI. However, while the AKI review showed that fluid accumulation and overload predicted mortality, this was not examined in other reviewed studies [23].

### 4.3 Implications

Overall, this review has demonstrated the significance of ED-AKI given its high incidence and poor outcomes. It calls for increased vigilance in ED physicians and need for further research. Currently, there is no formal definition for ED-AKI although this appears to be a neglected area that requires more focused attention. This also limits the potential for good reporting and analysis of ED-AKI to clarify its true epidemiology and outcomes.

Noting that the incidence of AKI was 20/1000 within 24 h of ED presentation which compares with 200/1000 within 48 h, early phases of AKI could have been missed when we remain reliant on blood creatinine levels for evaluating the acuity and kidney function status at the hospital front door. If this can be confirmed in clinical studies, it could indicate the need of other indicators for early assessment of kidney status. Risk factors identified, e.g. age, nursing home status, premorbid renal condition, and discharge diagnoses of diabetes, urological obstruction, sepsis, and gastrointestinal medical condition, may prompt ED physicians to consider AKI as a possible diagnosis in addition to other diagnosis. Red flags may be identified to prompt ED nurses and physicians to initiate early actions. At-risk patients should also be educated about the early signs and the need for prompt ED attendance.

This study also calls for a standardized protocol for initial investigations and management for patients identified as high risk for ED-AKI, e.g. standard method of objectively measuring urine output (such as a bladder scan or insertion of urinary catheter for urine output monitoring where clinically indicated); early correction of hypovolaemia; blood tests to assess renal function and electrolytes; urinalysis; and review of nephrotoxic medication. This may also lead way to developing risk prediction models for early identification of ED-AKI based on patient characteristics from triage. A multi-disciplinary approach is needed, including consultation with specially trained team members and nephrologists, pharmacists' input for screening drug-induced AKI, handover to general practitioners or specialists following the index episode, and post-discharge management and counselling. Risk factors of ED-AKI may differ slightly from those of general AKI or CA-AKI, but more research is needed since studies on ED-AKI is very limited. High hospital readmission rates may suggest a need for more thorough assessment of the patient's condition before discharge and follow-up post-discharge. Patients with risk factors for mortality may be monitored and managed more intensively to improve their outcomes.

### 4.4 Future research

The epidemiology and outcomes of AKI has been so extensively investigated that one may question whether there is a need for an ED focus i.e. ED-AKI. The UK NCEPOD has identified that lack of early recognition and appropriate management of hospital AKI are key factors for poor and preventable outcomes [16]. This review has shown that ED-AKI has a significant disease burden with a higher mortality rate compared to AKI. The front of the hospital and especially EDs are key areas of concern and relevance. The limited number of articles and information meeting the criteria for ED-AKI illustrates the neglect in this area. More evidence is needed to better understand ED-AKI epidemiology and outcomes. Particularly, studies identified were all conducted in high-income countries, which may limit their generalizability to tropical or low-income countries. Also, future research can examine the risk factors identified from the current review with adjustment for confounding.

## 5 Limitations

Despite the unique focus on ED-AKI and identification of studies with large sample size, this study has some limitations. First, only the PubMed database was used for the search of evidence due to the limited time and resources. Our search of PubMED was rigorous and systematic and followed all the principles for a systematic review with the exception of critically reviewing other available sources. Second, few studies met the inclusion criteria for the review. Some outcomes and risk factors were only reported in one study. Thus, a meta-analysis could not be performed. In the absence of large, high-quality, uniformly reported studies, we need to rely on general evidence on AKI studies. More research is needed to better understand the epidemiology of ED-AKI.

Studies identified were heterogeneous with variable findings. Study populations may differ in population demographics, and premorbid conditions, resulting in different incidences of AKI. Different studies also adopted different criteria for AKI definition. Thus, we cannot conclude if certain findings are repeatable. The high heterogeneity suggests that the pooled incidence may have obscured important underlying differences between the studies. Regarding risk factors for ED-AKI and post-ED-AKI mortality, there was no adjustment for confounder. Finally, only studies from high-income countries in Europe and Oceania could be identified. Thus, generalizability to low-income countries and countries in other parts of the world may be limited, especially since the aetiology of AKI tends to be different between tropical regions and low-income countries [25].

## 6 Conclusions

This systematic review reveals a paucity of relevant literature which calls for further research, increased vigilance, red flag identification, and standardized management protocols for ED-AKI. There is a high burden of AKI in the early, acute phase of hospitalized patients which we propose to define as ED-AKI. ED-AKI has an incidence of 20 per 1000 adult ED admissions in the first 24 h that increased to 200 per 1000 adult ED admissions in 48 h. The 24-h mortality rate was 4.56 %. Multiple important risk factors include increased age, nursing home residency, previous hospital admission within 30 days, higher environmental temperature, serum creatinine, bilirubin, C-reactive protein, white blood cell, alanine aminotransferase (ALT), and lower serum sodium and albumin; diabetes, urological obstruction, sepsis, and gastrointestinal medical condition, higher pre-admission mean creatinine, lower baseline eGFR, chronic kidney disease, prior history of AKI, lung disease, hyperlipidaemia, infections, antibiotics use.

## Data availability statement

Has data associated with your study been deposited into a publicly available repository? No.

This systematic review contains no new data other than a synthesis of findings from other original work. No unpublished data was received from authors of publications mentioned in this review. Our findings are all reported in the manuscript and supplementary material so there is no new data to deposit.

## CRediT authorship contribution statement

**Tsz Yan Cheung:** Writing – original draft. **Kelvin Lam:** Investigation. **Siu Chung Leung:** Conceptualization. **Timothy H. Rainer:** Conceptualization.

## Declaration of competing interest

The authors declare that they have no known competing financial interests or personal relationships that could have appeared to influence the work reported in this paper.
