# Supplementary material for: Clinical epidemiology and outcomes of emergency department-acute kidney injury: A systematic review
Source: Heliyon. 2024 May 4;10(9):e30580. doi: 10.1016/j.heliyon.2024.e30580 (PMC11096934; doi:10.1016/j.heliyon.2024.e30580)
Supplement: Multimedia component 1 [file mmc1.docx]

## Supplementary Information

### Supplementary I - Search Strategy

**Source Searched:** PubMed database (National Centre for Biotechnology Information, U.S. National Library of Medicine, National Institutes of Health, United States)

**Date of Search:** 14/8/2021 (all articles from 1996 to 14/8/2021)

**Search run by:** Tsz Yan Cheung

**Search Terms**

(((emergency department*[Title/Abstract]) OR (emergency service hospital[MeSH Terms]) OR (accident*[Title/Abstract] AND emergency department*[title/abstract]) OR (emergency room*[title/abstract]) OR (emergency medicine[Title/Abstract]) OR ("accident and emergency department*"[Title/Abstract]) OR ("A&E"[Title/Abstract]) OR ("A&Es"[Title/Abstract]) OR (ED[title/abstract]) OR (EDs[title/abstract])) AND ((acute kidney injury[MeSH Terms]) OR (acute kidney failure*[title/abstract]) OR (acute renal failure*[title/abstract]) OR (AKF[title/abstract]) OR (AKFs[title/abstract]) OR (ARF[title/abstract]) OR (ARFs[title/abstract]) OR (acute kidney injur*[title/abstract]) OR (acute renal injur*[title/abstract]) OR (AKI[title/abstract]) OR (AKIs[title/abstract]) OR (ARI[title/abstract]) OR (ARIs[title/abstract]) OR (acute renal insufficien*[title/abstract]) OR (acute kidney insufficien*[title/abstract]) OR (acute renal dysfunction*[title/abstract]) OR (acute kidney dysfunction*[title/abstract]) OR (AKD[title/abstract]) OR (AKDs[title/abstract]) OR (ARD[title/abstract]) OR (ARDs[title/abstract]) OR (acute kidney impair*[title/abstract]) OR (acute renal impair*[title/abstract]) OR (acute kidney disease*[title/abstract]) OR (acute renal disease*[title/abstract]) OR (early kidney failure*[title/abstract]) OR (early renal failure*[title/abstract]) OR (early kidney injur*[title/abstract]) OR (early renal injur*[title/abstract]) OR (early renal insufficien*[title/abstract]) OR (early kidney insufficien*[title/abstract]) OR (early renal dysfunction*[title/abstract]) OR (early kidney dysfunction*[title/abstract]) OR (AKD[title/abstract]) OR (early kidney impair*[title/abstract]) OR (early renal impair*[title/abstract]) OR (early kidney disease*[title/abstract]) OR (early renal disease*[title/abstract]) OR (acute kidney necrosis [title/abstract]) OR (acute kidney tubul* necrosis [title/abstract]))) AND ((((outcome*[Title/Abstract]) OR (prognosis[Title/Abstract]) OR (mortalit*[Title/Abstract]) OR (morbidit*[Title/Abstract]) OR (death*[Title/Abstract]) OR (survival*[Title/Abstract]) OR (complication*[Title/Abstract]) OR (length of stay[Title/Abstract]) OR (length of hospital stay[Title/Abstract]) OR (admission*[Title/Abstract]) OR (renal replacement therapy*[Title/Abstract]) OR (dialysis[Title/Abstract]) OR (haemodialysis[Title/Abstract]) OR (hemodialysis[Title/Abstract]) OR (renal transplant*[Title/Abstract]) OR (kidney ADJ3 transplant*[Title/Abstract]) OR (intensive care unit*[Title/Abstract]) OR (ICU[Title/Abstract]) OR (ICUs[Title/Abstract]) OR (intensive therapy unit*[Title/Abstract]) OR (intensive treatment unit*[Title/Abstract]) OR (critical care unit*[Title/Abstract]) OR (chronic kidney failure*[Title/Abstract]) OR (chronic kidney injur*[Title/Abstract]) OR (chronic kidney disease*[Title/Abstract]) OR (CKD[Title/Abstract]) OR (CKDs[Title/Abstract]) OR (CKF[Title/Abstract]) OR (CKFs[Title/Abstract]) OR (CKI[Title/Abstract]) OR (CKIs[Title/Abstract]) OR (mortality[MeSH Terms]) OR (morbidity[MeSH Terms]) OR (renal replacement therapy[MeSH Terms]) OR (hospitalization[MeSH Terms]) OR (critical care[MeSH Terms]) OR (morbidity[MeSH Terms]) OR (renal insufficiencies, chronic[MeSH Terms])) OR ((Risk Factors[MeSH Terms]) OR (Comorbidity[MeSH Terms]) OR (risk factor*[Title/Abstract]) OR (aetiolog*[Title/Abstract]) OR (associat*[Title/Abstract]) OR (comorbidit*[Title/Abstract]) OR (co-morbidit*[Title/Abstract]) OR (Pharmaceutical Preparations[MeSH Terms]) OR (Infections[MeSH Terms]) OR (Institutionalization[MeSH Terms]) OR (drug*[Title/Abstract]) OR (medicine*[Title/Abstract]) OR (medication*[Title/Abstract]) OR (prescription*[Title/Abstract]) OR (herb*[Title/Abstract]) OR (infect*[Title/Abstract]) OR (sepsis[Title/Abstract]) OR (old age home*[Title/Abstract]) OR (institution*[Title/Abstract]) OR (retirement home*[Title/Abstract]) OR (nursing home*[Title/Abstract]) OR (old aged home*[Title/Abstract]))) OR ((epidemiology[MeSH Terms]) OR (epidemiology[Title/Abstract]) OR (epidemiologic studies[MeSH Terms]) OR (epidemiologic stud*[Title/Abstract]) OR (incidence*[Title/Abstract]) OR (demograph*[Title/Abstract]) OR (prevalence*[Title/Abstract])))

### Supplementary Table 1 - Critical Appraisal

|  | Woitok, B. K., et al. (2020). "Dysnatremias in emergency patients with acute kidney injury: A cross-sectional analysis." Am J Emerg Med 38(12): 2602-2606. | Foxwell, D. A., et al. (2020). "Epidemiology of emergency department acute kidney injury." Nephrology (Carlton) 25(6): 457-466. | Xu, Z., et al. (2020). "Heat and risk of acute kidney injury: An hourly-level case-crossover study in Queensland, Australia." Environ Res 182: 109058. | Stucker, F., et al. (2017). "Risk factors for community-acquired acute kidney injury in patients with and without chronic kidney injury and impact of its initial management on prognosis: a prospective observational study." BMC Nephrol 18(1): 380. | Stack, A. G., et al. (2020). "Temporal trends in acute kidney injury across health care settings in the Irish health system: a cohort study." Nephrol Dial Transplant 35(3): 447-457. | Jonsson, A. J., et al. (2019). "Computerized algorithms compared with a nephrologist's diagnosis of acute kidney injury in the emergency department." Eur J Intern Med 60: 78-82. | Soto, K., et al. (2016). "The risk of chronic kidney disease and mortality are increased after community-acquired acute kidney injury." Kidney Int 90(5): 1090-1099. |
| --- | --- | --- | --- | --- | --- | --- | --- |
| 1. Was the research question or objective in this paper clearly stated? | Yes | Yes | Yes | Yes | Yes | Yes | Yes |
| 2. Was the study population clearly specified and defined? | Yes | Yes | Yes | Yes | Yes | Yes | Yes |
| 3. Was the participation rate of eligible persons at least 50%? | Yes | Yes | Yes | Yes | Yes | Yes | Yes |
| 4. Were all the subjects selected or recruited from the same or similar populations (including the same time period)? Were inclusion and exclusion criteria for being in the study prespecified and applied uniformly to all participants? | Yes | Yes | Yes | Yes | Yes | Yes | Yes |
| 5. Was a sample size justification, power description, or variance and effect estimates provided? | Yes | Yes | Yes | Yes | CD | No | Yes |
| 6. For the analyses in this paper, were the exposure(s) of interest measured prior to the outcome(s) being measured? | NA | Yes | Yes | Yes | CD | Yes | Yes |
| 7. Was the timeframe sufficient so that one could reasonably expect to see an association between exposure and outcome if it existed? | NA | Yes | Yes | Yes | NA | Yes | Yes |
| 8. For exposures that can vary in amount or level, did the study examine different levels of the exposure as related to the outcome (e.g., categories of exposure, or exposure measured as continuous variable)? | NA | Yes | Yes | Yes | NA | Yes | Yes |
| 9. Were the exposure measures (independent variables) clearly defined, valid, reliable, and implemented consistently across all study participants? | NA | Yes | Yes | Yes | CD | Yes | Yes |
| 10. Was the exposure(s) assessed more than once over time? | NA | No | Yes | No | NA | No | No |
| 11. Were the outcome measures (dependent variables) clearly defined, valid, reliable, and implemented consistently across all study participants? | Yes | Yes | Yes | Yes | Yes | Yes | Yes |
| 12. Were the outcome assessors blinded to the exposure status of participants? | NA | No | NR | NR | NR | NR | NR |
| 13. Was loss to follow-up after baseline 20% or less? | Yes | Yes | NA | No | NA | NR | No |
| 14. Were key potential confounding variables measured and adjusted statistically for their impact on the relationship between exposure(s) and outcome(s)? | No | Yes | NA | Yes | CD | No | Yes |
| Quality Rating | Fair | Good | Good | Fair | Fair | Fair | Good |
| CD = Cannot determine, NA = Not applicable, NR = Not reported | | | | | | | |
